# Analysis, identification and visualization of subgroups in genomics

**DOI:** 10.1093/bib/bbaa217

**Published:** 2020-09-21

**Authors:** Gunnar Völkel, Simon Laban, Axel Fürstberger, Silke D Kühlwein, Nensi Ikonomi, Thomas K Hoffmann, Cornelia Brunner, Donna S Neuberg, Verena Gaidzik, Hartmut Döhner, Johann M Kraus, Hans A Kestler

**Affiliations:** Institute of Medical Systems Biology (MSB), Ulm University, Ulm, Germany; Department of Otorhinolaryngology, Head and Neck Surgery, Ulm University Medical Center, Germany; MSB and deputy of Core Unit Bioinformatics, Ulm University, Ulm, Germany; International Graduate School of Molecular Medicine, Ulm University, Germany; International Graduate School of Molecular Medicine, Ulm University, Germany; Department of Otorhinolaryngology, Head and Neck Surgery, Ulm University Medical Center, Germany; Department of Otorhinolaryngology, Head and Neck Surgery, Ulm University Medical Center, Germany; Department of Biostatistics, Dana-Farber Cancer Institute, Boston, Massachusetts, USA; Department of Internal Medicine III, Ulm University Medical Center, Germany; Department of Internal Medicine III, Ulm University Medical Center, Germany; MSB, Ulm University, Germany; Institute of Medical Systems Biology, and head of Core Unit Bioinformatics, Ulm University, Ulm, Germany

**Keywords:** visualization, exploratory analysis, multi-objective optimization, vaccination targets

## Abstract

**Motivation:**

Cancer is a complex and heterogeneous disease involving multiple somatic mutations that accumulate during its progression. In the past years, the wide availability of genomic data from patients’ samples opened new perspectives in the analysis of gene mutations and alterations. Hence, visualizing and further identifying genes mutated in massive sets of patients are nowadays a critical task that sheds light on more personalized intervention approaches.

**Results:**

Here, we extensively review existing tools for visualization and analysis of alteration data. We compare different approaches to study mutual exclusivity and sample coverage in large-scale omics data. We complement our review with the standalone software AVAtar (‘analysis and visualization of alteration data’) that integrates diverse aspects known from different tools into a comprehensive platform. AVAtar supplements customizable alteration plots by a multi-objective evolutionary algorithm for subset identification and provides an innovative and user-friendly interface for the evaluation of concurrent solutions. A use case from personalized medicine demonstrates its unique features showing an application on vaccination target selection.

**Availability:**

AVAtar is available at: https://github.com/sysbio-bioinf/avatar

**Contact:**

hans.kestler@uni-ulm.de, phone: +49 (0) 731 500 24 500, fax: +49 (0) 731 500 24 502

## Introduction

High-throughput biomolecular technologies make multi-modal data available for diverse biological and medical settings. Analysis and visualization of genomic and transcript-omic maps are important steps not only for illustration but also for exploratory analysis [35, 45, 46, 70, 86]. In this context, computer assistance is paramount in the objective analysis of data such as gene mutations or overexpression of genes [74]. Most importantly, intuitive visualization tools simultaneously integrating different data modalities are required [87]. These data modalities such as mutation, expression or methylation profiles can be depicted as alteration plots, comparing patients’ coverage for the examined alteration to alteration exclusivity for the single patient’s sample.

Commonly, alteration plots are created manually by researchers in time-consuming processes, although specialized analysis tools for alteration data may include basic visualization functionality, e.g. cBioPortal [14, 30], Gitools [79], UCSC Cancer Genomics Browser [85], Integrative Genomics Viewer [84], IntOGen [32], MAGI [57] and caOmicsV [117].

Visualization and annotation of genomic alteration are only the first steps in deepening the knowledge on disease development and progression. Therefore, a huge effort has also been made in the context of the analysis of genome alteration data. Detection of mutually exclusive alterations has been shown to provide crucial information in the context of cancer development and investigation of therapeutic approaches, also in light of personalized treatments [24]. Due to the extensive heterogeneity in cancer genomes, most patients possess only a single driver mutation [105]. Hence, groups of genes harboring driver mutations tend not to co-occur in the same sample, as also shown in different cancer cohorts [11, 15]. Many cancer-related genes are involved in the phenomenon of mutual exclusivity [28]. Exemplarily, BRAF and NRAS, both members of the MAPK pathway, are widely altered in patients with melanoma [9], thyroid carcinoma [25], myeloma [71] and colorectal cancer [64]. However, few patients harbor both alterations [9]. Strikingly, the forced expression of two mutually exclusive genes in lung adenocarcinoma [101], KRAS and EGFR, causes proliferation and survival disadvantage to cancer cells [41]. The biologically motivated hypothesis behind mutual exclusivity is based on either functional redundancy [15, 17, 21, 90, 109] of these genes or synthetic lethality [16, 27]. Hence, having efficient algorithms to subgroup genes based on their mutual exclusivity gives both insights on patients’ specific sub-groupings and potential personalized therapeutic interventions.

**Figure 1:**
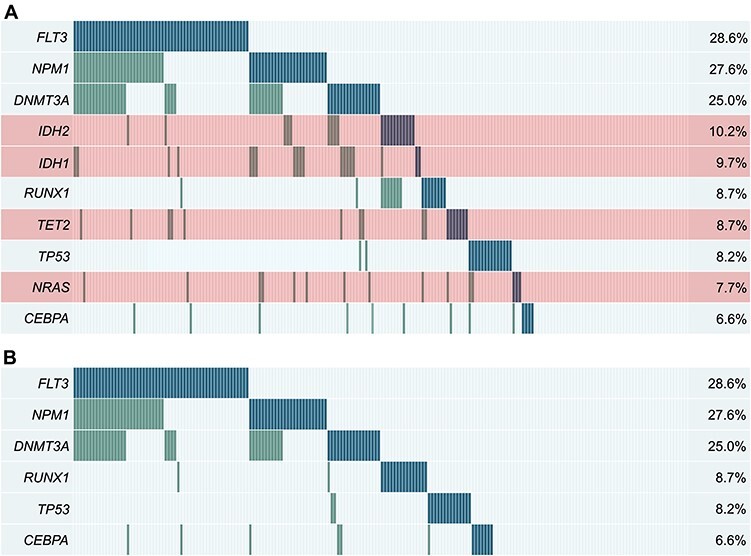
Alteration plots for gene selections of the TCGA AML dataset [99]. Every row represents a gene and every column a sample. If a sample has an alteration in a gene, the corresponding cell is marked (blue: first alteration in sample, green: overlapping alterations). (A) Top 10 genes with most frequent mutations sorted by sample coverage. (B) Example of optimization of the gene set selection with six genes (sorted by sample coverage). Genes highlighted in A (red) have been excluded by the optimization of the gene set selection.

As an illustrative example, the TCGA AML (acute myeloid leukemia) dataset [99], containing mutation data of 200 samples, is used. Figure 1A, shows the alteration plot for the top 10 genes with the highest sample coverage. In the figure, each patient sample is a column of the plot, whereas every row represents a gene that can be altered or not in each sample. Coverage is defined as the representativeness of alteration within all samples. The overlap is considered as the co-occurrence of multiple mutations within the same sample. In Figure 1 considerable overlap (less mutually exclusivity) between the genes can be observed. Here, to further optimize the gene set selections, different algorithms can be applied. The final aim is to find subsets of genes with high coverage together with high mutual exclusivity (Figure 1B). These approaches are based either on alteration data only (*de novo*) or integration with experimental knowledge or databases (knowledge-based approaches).

In the following, we will review available visualization and analysis tools for gene alterations. Besides one exception, none of the currently available visualization tools provides implementations for gene drivers selection. Hence, we introduce our software ‘analysis and visualization of alteration data’ (AVAtar) that incorporates both visualization and analysis approaches. AVAtar has unique features for import, filtering and a combination of private and database data together with numerous export functions. Different from all other analysis software, AVAtar provides a user-friendly interface suitable for life scientists without the need for programming skills. To explore the possibilities provided by AVAtar, we first compared its features to available tools for both visualization and analysis. Second, we summarized a use case from an already published study [29] regarding semi-personalized selection of candidate vaccination targets for head and neck squamous cell carcinomas (HNSCC).

## Genomic analyses to semi personalized medicine

Since the first human genome has been made available [18], the investigation and interpretation of genomic data have been a focus of modern molecular biology [53]. Technical improvements and strong reduction of sequencing costs have permitted genomic information to be more and more included in medical practice [10]. Thanks to introduction of genomic analyses, cancers can now be defined by their molecular drivers. This is not only useful in cancer identification but also for treatment decisions [10]. In fact, some traditional treatments could be proved ineffective or have side effects in certain patient populations [10, 98]. For example, tamoxifen was long time used to treat breast cancer patients. However, nowadays it is known that patient-specific alterations affecting its active metabolite exist [37]. Hence, analyses of genomic alterations is an important step towards semi-personalized medicine. Moreover, minimal sets of intervention targets might be of interest.

## Visualization tools

Tools for visualization of genomic data are relevant for understanding and describing connections between genomic alterations and cancer [76, 88, 110]. Different approaches for visualization have been suggested (Table 1), such as genomic coordinate views, heat maps, network views, aberration plots and transcript views. The advantages of genomic coordinates-based tools rely on the possibility of accessing detailed sequences and various types of alterations. However, they can display limited numbers of samples and genes simultaneously [117]. Examples of these tools are cBioPortal [14, 30], Integrated Genomics Viewer [84], MAGI [57] and USC Cancer Genome Browser [85]. Besides genomic coordinate view approaches, heat maps and network views allow visualization of multiple genomic alterations in broad groups of genes and samples [14, 108, 114]. Hence, for further analyses, these types of genomic visualization are preferred. In accordance, most of the available tools include heat map visualization. A distinguishing feature of these tools is the possibility of combining private and database data, as well as exporting both raw data and figures (Table 1). In this regard, AVAtar is the only tool that provides a complete set of features for both import and export.

**Table 1:** Available visualization tools for genomic alterations. For each tool, its application type (R package, Web application with local installation or standalone software) is specified as well as analysis, visualization, import and export features. A $\checkmark $ indicates that the feature is available, ‘−’ indicates it is not present

**Name**	**Application type**	**Analysis/visualization**				**Import**			**Export**	
		Alteration plots	Pathway subgrouping	Gene-set selection	Dataset preprocessing	Databases	Privatedata	Combined data	Raw data	Figures
caOmicsV [117]	R package	✓	–	–	–	–	✓	–	✓	✓
cBioPortal [14, 30]	Web/local	✓	✓	✓	–	✓	✓	–	✓	✓
Gitools [79]	Web/local	✓	–	–	–	✓	✓	–	✓	✓
Integrated Genomics Viewer [84]	Web/local	✓	–	–	✓	✓	✓	✓	–	–
IntOGen [32]	Web/local	✓	✓	✓	–	✓	✓	–	✓	–
MAGI [57]	Web/local	✓	✓	–	✓	✓	✓	✓	–	✓
UCSC Cancer Genome Browser [85]	Web/local	✓	✓	–	✓	✓	✓	✓	–	–
AVAtar	Standalone	✓	✓	✓	✓	✓	✓	✓	✓	✓

## Tools for gene selection of mutual exclusivity

**Figure 2:**
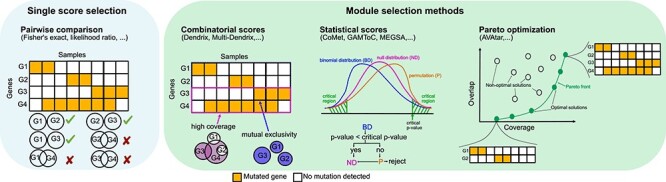
*De novo* approaches overview for investigation of mutual exclusivity. On the left blue box, single score selection is depicted. It is performed by applying pairwise test on alteration data. On the right green box, module selection methods are illustrated. Sets of mutually exclusive genes are selected by different approaches: combinatorial scores, statistical scores and Pareto optimization.

Different computational approaches have been developed to investigate driver gene mutations. They are mainly divided into *de novo* and knowledge-based approaches, where experimental information is integrated into the algorithms. Even if many tools for knowledge-based investigation are available [2, 5, 7, 8, 12, 13, 15, 31, 34, 38, 40, 47, 48, 59, 61, 65, 78, 80, 83, 92, 96, 97, 100, 113, 116, 118, 119], the fact that they require information on either pathways, interaction networks, or functional phenotypes data makes their broad application limited. Hence, *de novo* methods will be the focus of this review. In general, *de novo* methods are based only on alteration data. Two main strategies for the selection of mutually exclusive genes have been classically applied (Figure 2). One of the simplest approach to investigate mutual exclusivity is pairwise statistical analyses such as Fisher’s exact test or likelihood ratio methods [24]. However, this approach has many limitations. First, it assumes that genetic alterations are evenly distributed across samples, which does not face the reality of alteration data [1, 3, 51, 54]. This problem was addressed by the WeSME approach that includes a weighted sampling proportional to the observed mutation frequency [49]. In addition, mutual exclusivity frequently does not involve only a few genes [115], making the pairwise test approach not suitable for investigating modules [67]. For this purpose, algorithms searching for modules of mutually exclusive mutations have been developed. Here, the new addressed task is to find sets of genes whose alterations cover high number of samples (coverage) together with low number of overlapping alterations in the set (high mutually exclusivity). This problem has been addressed from different perspectives. The combinatorial score approach, such as the Dendrix and its extension [56, 106], uses greedy algorithms to maximize the combination of these two objectives by expanding a seed set of genes. However, this method that tries to maximize both coverage and exclusivity can be biased towards high frequencies genes sets [105]. For this reason, other modules selection approaches have been implemented. Exemplarily, CoMet [60], MEGSA [77], and GAMToc [68] focus on selecting modules based on statical significance instead of maximization of scores (Figure 2). Another approach to overcome the problems behind score maximization has been implemented in AVAtar. Instead of defining weights a priori and searching for a single optimal gene selection, a set of gene selections consisting of the optimal trade-offs (defined as Pareto set) between the objectives can be identified (Figure 2). This allows the researcher to interactively explore the optimal trade-offs found and to choose gene sets based on task-specific background knowledge. 

Another limitation of available tools to compute mutual exclusivity is their running environment (Table 2). Almost all tools require programming and bioinformatics skills of the user, thus excluding a wide range of life science researchers. On the other hand, AVAtar provides user-friendly graphical interface. In this context, also cBioPortal [14, 30] presents the possibility to perform mutual exclusivity analysis with a user-friendly graphics. However, the algorithm is based on pairwise tests that have already been shown to be quite limited [14, 24, 30]. Instead, AVAtar implements a modular search based on Pareto set optimization. Moreover, the user can preprocess data and combine sets of private and database searches in a unique form.

### Multi-objective evolutionary algorithm implemented in AVAtar

Weighting the importance of contradicting aims is a common approach to resolve conflicts and allows the application of standard optimization algorithms. However, there is neither a generally applicable weighting for all datasets nor an obvious and objective weighting for a given dataset that guarantees an optimal selection. Here, multi-objective optimization [22] offers an alternative approach. Technically, the task consists in finding a subset of genes ${G}$ from a given set of genes ${{\mathcal{G}}}$ (${G} \subseteq{{\mathcal{G}}}$) such that the number of covered samples ${\gamma } ({G})$ is maximized and either the overlap ${\omega }({G})$ or the number of genes $|{G}|$ is minimized. This introduces a multi-objective optimization problem that results in finding a Pareto-optimal set ${{{\mathcal{S}}}^*} \subseteq{{\mathcal{S}}}$ of gene subsets within the set of all gene subsets ${{\mathcal{S}}} = { {G} \subseteq{{\mathcal{G}}}}$. To this purpose, we developed an evolutionary algorithm for the multi-objective gene selection task based on the Non-dominated Sorting Genetic Algorithm II [22, NSGA-II] (implemented by jMetal library v.5.3 [73]). This is a population-based metaheuristic that adapts concepts of the theory of evolution [81]. A set of solutions, called population, is evolved iteratively by applying recombination and mutation operators to the solutions. Finally, AVAtar facilitates the creation of objective and reproducible alteration plots by offering algorithmic sorting of genes and samples. The task of finding a gene order based on the additionally covered samples is formulated as a minimal set cover problem. A modified greedy algorithm [20] for set covering is applied [44]. Starting with an initial solution, the greedy algorithm incrementally adds the gene covering the most uncovered samples to its current partial solution. Further details of the algorithm are described in the Supplementary Information.

**Table 2:** Available software for identification of mutually exclusive genes in alteration data. For each of the tools, the method used to identify mutually exclusive alterations is stated. At last, running environment needed is reported. Tools marked by an asterisk (*) have both visualization and analysis features

Name	Method	Running environment
cBioPortal [14, 30]^*^	Pairwise test	Web tool
CoMET [60]	Statistical score	Python
Dendrix [106]	Combinatorial scores	Python
GAMToc [68]	Statistical score (entropy score)	Matlab
MEGSA [77]	Statistical score	R
Multi-Dendrix [56]	Combinatorial score	Python
MutExSL [94]	Pairwise test	Excel
RME [23]	Combinatorial score	Bash
TiMEX [19]	Statistical score	R
WeSME [49]	Stastical score	Python
WExT [58]	Statistical score	Python
AVAtar^*^	Pareto front	Standalone software

**Figure 3:**
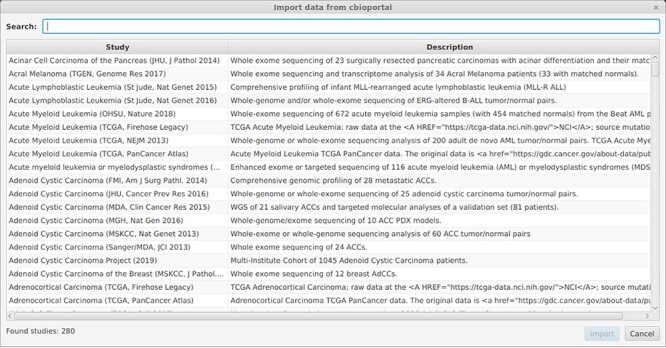
Dialog for selecting a study to import from cBioPortal. Studies can be searched by specifying terms that occur in their id, name or description.

## AVAtar

The multi-objective approach using a Pareto front was integrated in a readily usable standalone software AVAtar. There are no additional software requirements. After the extraction of the downloaded AVAtar archive, the user can immediately start to use it. The project repository of AVAtar available at https://github.com/sysbio-bioinf/avatar includes a detailed user manual with a stepwise walkthrough describing the application of AVAtar in the HNSCC analysis. The walkthrough already underlines our user-friendly interface, suitable for life scientists. AVAtar supports the import from different data sources: data files (Excel, Text), cBioPortal [14, 30] and Gene Expression Omnibus [6]. The import dialog with builtin search capabilities is shown in Figure 3. It is possible to combine data from multiple studies and to integrate different alteration types. Clinical attributes can be imported to define sample groups for analysis and visualization. Moreover, AVAtar’s optimization setup is designed to offer default algorithm parameter values that are suitable for a broad range of applications. A batch mode to perform multiple optimizations in parallel on a compute server is available. It can be started as follows:

java11 -jar avatar.jar -f hnsc.avatar -o usecase.batch -t 6 Runs: 0/7 - Progress: 0.243% - Estimated Duration: 05:42:54

Resulting gene selection sets can be explored interactively as alteration plots and grouped by functional categories. Additionally, graphics contrasting alternative gene selections can also be created. Finally, all graphics obtained can be exported as publication ready vector graphics.

### Comparison between AVAtar and the Multi-Dendrix approach

**Figure 4:**
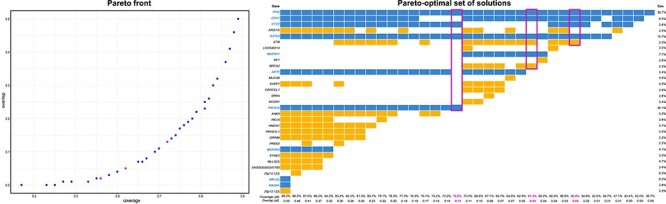
Visualization of the Pareto set resulting from a coverage maximization and overlap minimization on the breast cancer dataset used to evaluate the Multi-Dendrix algorithm [55]. On the left, the Pareto front is represented. Each point in the Pareto front represents a gene set solution for a certain coverage–overlap combination. Magenta dots represent the highlighted solution of the right figure. On the right, the corresponding Pareto set solutions for gene selection are depicted. Each column represents a solution of the Pareto set and each row contains a gene. The occurrence of a gene in a solution is marked by a colored rectangle. The solutions found also by the Multi-Dendrix algorithm are marked in blue, whereas new genes idenfied by AVAtar are marked in orange. The three selected solutions highlighted in magenta are shown in Figure 5.

**Figure 5:**
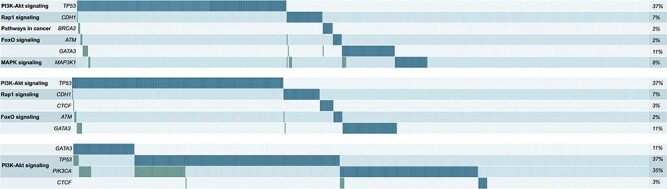
Mutation plot of the four gene solution of the Pareto set of Figure 4 with most promising gene set selections.

Deng *et al.* [24] already performed a benchmark comparison among a variety of available algorithms for mutual exclusivity investigation. From their analysis, the Multi-Dendrix algorithm [56] performed best. Hence, we compared the performance of AVAtar to this approach. We used the breast cancer dataset of the Multi-Dendrix publication [56]. The Multi-Dendrix algorithm uses a fixed a priori weight between coverage and overlap to find a specified small number of pairwise disjoint sets of genes with mostly mutually exclusive mutations within the sets. The genes of the found sets are considered as potential driver genes. The multi-objective evolutionary algorithm of AVAtar does not weight coverage and overlap of the gene sets but instead searches for the set of optimal tradeoffs between these two objectives. In Figure 4, the Pareto front and the corresponding set of selected genes resulting from our analysis are shown. Cells colored in blue are the genes also identified from the Multi-Dentrix. Genes with higher coverage are identified by both methods and are also represented in most of our solutions. In orange, we depicted new genes found by AVAtar. First, it can be observed that part of new hits is represented in solution with high coverage and overlap. However, we highlighted some interesting sets found by combinations of good coverage and low overlap (magenta rectangles). For these solutions, we provide the corresponding alteration plots in Figure 5. Moreover, similarly to the Multi-Dendrix approach, we provided also pathway subgrouping information. In these sets, AVAtar identified crucial genes for breast cancer as ATM [26, 33, 36, 66, 69, 112] and BRCA2 [42, 63, 72, 93, 102, 104] that were not found by the Multi-Dendrix approach. ATM is selected together with TP53 in two of our highlighted solutions. This supports our approach since ATM is widely reported to be mutually exclusive with TP53 in breast and also other types of cancers [39, 82, 91, 111]. Moreover, we could also select smaller sets with higher coverage on patients samples (72%). This set is better than the best set selected by the Multi-Dendrix approach in terms of coverage, overlap, and the number of genes selected. Thus, AVAtar empowers the user by providing all possible combination of the best trade-offs between coverage and mutual exclusivity. This possibility is of great relevance given that there is no commonly shared method to set a weight between these two conflicting objects.

**Figure 6:**
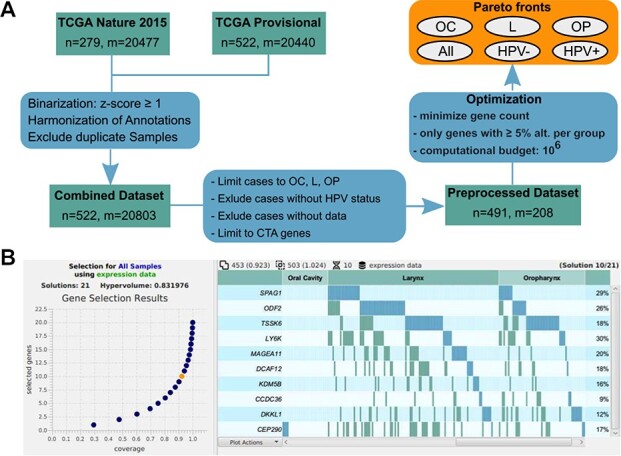
Preparation of the HNSCC analysis and AVAtar optimization result. (A) Analysis workflow for the HNSCC use case: data preparation steps and optimization settings. (B) Pareto set dialog of AVAtar. The found Pareto set (21 solutions) is shown on the left and a plot of the selected solution (orange circle) is shown on the right.

## Use case: vaccination targets for HNSCCs

In the following sections, we will further guide through the features of AVAtar by presenting the use case also available on the tutorial of the software. An in-depth medical description of the analysis performed on AVAtar can be found in [29]. The HNSCC analysis aims is to compare promising vaccination target sets for different subgroups of patients with HNSCC. Clinically, HNSCC shows distinct survival differences between the main three primary tumor sites: oral cavity (OC), oropharynx (OP) and larynx (L) [52]. Furthermore, HNSCC can be divided based on the main drivers of carcinogenesis: noxious agents (smoking, alcohol) or high-risk human papillomaviruses (HPV) [43, 107]. HPV-positive HNSCC is characterized by a much better prognosis compared to HPV-negative HNSCC, which has previously been shown for multiple treatment strategies [4, 62]. Shared cancer antigens, in particular cancer-testis antigens (CTA), could play an important role in future immunotherapy strategies for both HPV-negative and HPV-positive HNSCC [52, 95, 103].

Thus, a comprehensive analysis of the CTA repertoire as model antigens is needed to identify which antigens to target based on primary tumor site and HPV status. To rationalize vaccination efforts in clinical trials and to avoid the need for cumbersome individual testing of antigen expression, a semi-personalized off-the-shelve multi-antigen vaccine covering a high rate of the respective patient cohort is desired. In particular, for specific vaccination strategies, the number of target antigens that can reasonably be combined within one vaccine is limited. The task to find vaccination targets is formulated as multi-objective gene selection. Here, we applied coverage maximization and subset size minimization.

### Dataset preparation and optimization setup for HNSCC analysis

AVAtar offers the possibility to pre-process data by deleting samples not relevant for the desired analysis, grouping samples according to clinical attributes, or filtering desired genes. More sophisticated preprocessing based on clinical data (e.g. harmonization of primary sites) can be accomplished by exporting the clinical data. Such editing and harmonization of clinical annotations are unique features of AVAtar.

In the specific use case, two publicly available large datasets of HNSCC patients (TCGA 2015 [75], TCGA provisional) have been combined for the analysis. The clinical annotation for HPV status is well defined in TCGA 2015 ($>1000$ HPV E6/E7 RNA reads) but in TCGA provisional a surrogate marker for HPV-association was used (p16 immunohistochemistry). Therefore, the two datasets have been combined to obtain the well-defined HPV-status definition for the TCGA 2015 subset of patients. In our use case, the datasets have been downloaded on 25 October 2018. By accessing the preprocessing features of AVAtar cited above, the combined dataset has been prepared as follows (see also Figure 6A):

1. The expression data from the dataset TCGA 2015 have been inserted with alterations defined as overexpression using a threshold equal to the standard deviation.
2. The expression data from the dataset TCGA provisional ($522$ samples with expression profiles) have been imported with alterations defined as overexpression using a threshold equal to the standard deviation. Duplicate samples that are part of TCGA 2015 are excluded resulting in a total of 522 samples.
3. The primary tumor sites (clinical data) have been grouped and renamed for compliance with the other dataset. This has yielded the primary sites oral cavity, oropharynx, larynx and hypopharynx.
4. The HPV status (attribute HPV STATUS) for the samples from TCGA provisional has been determined based on the HPV-p16-status (attribute HPV STATUS P16) and primary site.
5. The samples have been grouped by the primary site. Samples with primary site lip ($m=2$) and hypopharynx ($m=10$) have been deleted.
6. The samples without an assigned HPV status ($m=19$) have been deleted as well.
7. Genes without overexpression and non-CTA genes have been deleted.

**Figure 7:**
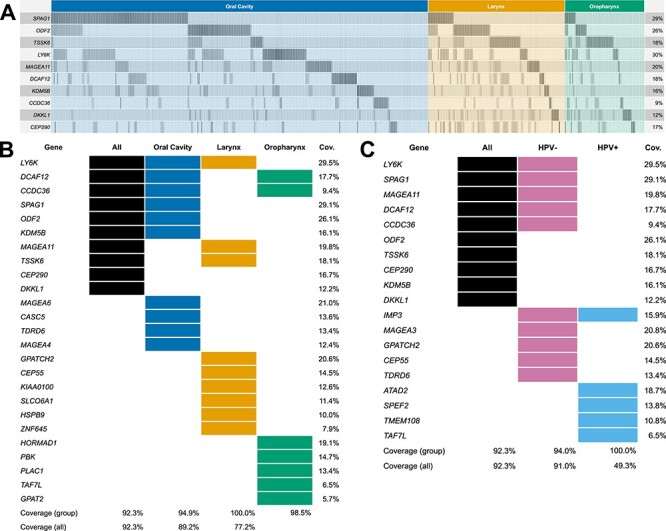
Optimization results for the HNSCC analysis. The objectives *maximal coverage* and *minimal number of genes* have been used in the optimization. (A) Alteration plot (overexpression) of the 10-gene solution resulting from the optimization for all samples. Clinical subgroups are shown. (B+C) The solution comparison tables show which genes are part (colored rectangles) of which solution (column). The solutions with at most 10 genes per primary site (B) and per HPV-status (C) are compared to the 10-gene solution for all samples.

The resulting dataset has $491$ samples and $208$ genes. Further, the user can proceed to perform a gene analysis to get mutually exclusive gene alterations within the selected and visualized subgroups. In our use case example, we ran the optimization with coverage maximization and gene count minimization on all samples and on the selected sample groups: oropharynx, larynx, oral cavity, HPV+ and HPV-. For the optimization of each sample group, we chose to consider only the genes with at least $5\%$ alterations within that sample group. Optimization parameters for the HNSCC analysis are listed in Table 3. A fine-grained search with two swapped genes in expectation is used. This is compensated by a larger number of search steps ($10^6$). Due to the large computation budget of $10^6$ iterations with $100$ solutions resulting in $10^8$ solution evaluations, the batch optimization mode of AVAtar has been used to perform the six optimization runs in parallel on a compute server.

### Result visualization

An optimization using AVAtar has been performed for the whole cohort and the sample groups resulting from the clinical attributes of the primary tumor site and HPV status. For each Pareto-optimal set, the solution with the largest coverage and at most 10 genes have been selected. For the optimization within the whole cohort, a 10-gene solution with a coverage of $92.3\%$ has been found (Figure 6B). The visualization of coverage and overlap by the primary site is displayed in Figure 6B and Figure 7A. However, the gene set optimized for coverage of all patients is dominated by OC patients, since these patients represent $64\%$ of the cohort. This 10-gene set has coverage of $91.7\%$ among OC, $96.5\%$ among L and only $87.8\%$ among OP indicating molecular differences among these primary sites. The optimizations for the different primary sites yield distinct gene sets (Figure 7B). Comparing these semi-personalized selections, it becomes evident that a selection of up to 10 genes optimized for the respective group of primary tumors results in an optimal coverage for the respective group with a suboptimal coverage in other primary sites. Since the OP cohort consisted primarily of HPV-positive patients ($72.7\%$) in contrast to the other primary sites, this leads to the hypothesis that the main differences in the CTA repertoire may be due to HPV status. Optimizing for HPV status and comparing the gene selections of up to 10 genes for HPV-positive and HPV-negative patients, a distinct gene selection overlapping only in one gene can be observed (Figure 7C). The 10-gene selection found for the HPV-negative patients differs from the selection for all patients in five genes. The five-gene selection found for the HPV-positive patients contains completely distinct genes compared to all-patients selection and has only one common gene with the HPV-negative selection. An in-depth medical discussion of the obtained results can be found in [29].

**Table 3:** Optimization parameter setup of the use case. The parameters are given as the easier interpretable values from the ‘simple setup’ in AVAtar (second column) and the corresponding values from the algorithm description (third column)

Parameter	Value	Algorithm parameter value
Population size	$100$	$\mu = 100$
Solution combination count	$5$	${p_{\textrm{cx}}} = 0.1$
Initially selected genes	$20$	${p_{\textrm{sel}}} \approx 0.0962$
Swapped genes	$2$	${p_{\textrm{mut}}} \approx 0.0096$
Selection pressure	$10$	${\tau } = 10$
Search steps	$10^6$	$k = 10^6$

## Conclusion

Nowadays, visualization and selection of gene alterations in large datasets are central issues in cancer research. In particular, in the context of providing intervention targets for personalized medicine approaches. Herewith, we revised available visualization and analysis tools and present AVAtar, our comprehensive software for gene visualization that also tackles the issue of target selection. An evolutionary algorithm is built into AVAtar to find trade-off solutions, which then can be explored interactively. Here, finding optimal gene subsets such as target selection for semi-personalized vaccination is formulated as a multi-objective optimization task. We further presented a walkthrough for the use of AVAtar by showing a real case scenario already applied in medical research [29]. The analysis of HNSCC expression data demonstrates the capabilities of AVAtar focusing on data import, visualization and optimization. In this context, we could show that subgroup-focused analysis can be performed with AVAtar by applying the optimization algorithm on different patient groups separately. If the optimization is executed separately for different clinical or molecular patient groups, distinct optimal gene selections become evident underlining the importance of subgroup-focused analyses in clinical trials. The diversity in the selected genes depending on the considered subgroups is in line with distinct molecular differences between HPV-positive and HPV-negative HNSCC [43, 50, 89]. Apart from the demonstrated use case, AVAtar can be used for explorative data analysis on binary or binarizable data, e.g. mutation, expression and methylation data. The interactive exploration of the trade-offs for the gene selection problem found by the optimization algorithm is a unique feature of AVAtar. To the best of our knowledge, AVAtar is the first software integrating visualization of alteration data and analysis via multi-objective optimization in an easily operable graphical user interface. This is complemented by the cBioPortal and Gene Expression Omnibus import functionality that provides access to a vast amount of published data. Finally, the gene selection optimization is a general method, which can be used for further research questions such as optimal gene selection for panel sequencing.

**Key Points**

- Overview of visualization and analysis tools for gene alterations in cancer with features and limitation. Potential improvement in the analysis and visualization of gene alterations.
- Introduced a comprehensive platform that integrates diverse aspects for gene alteration visualization and analysis. AVAtar provides a user-friendly interface that offers unique data-set processing features, gene selection analysis with an already implemented algorithm for multi-objective gene selection and exportable results.
- AVAtar was successfully applied to identify candidate vaccination targets for HNSCC in different sub-groups of patients.

## Supplementary Material

Supplement_algorithm_bbaa217

## Data Availability

Datasets are provided on TCGA (https://portal.gdc.cancer.gov/projects/TCGA-LAML) and cBioPortal (http://www.cbioportal.org/study/summary?id=hnsc_tcga). AVAtar code and walkthrough is available at GitHub (https://github.com/sysbio-bioinf/avatar).

## Notes

**Gunnar Völkel** is a postdoctoral researcher at the Institute of Medical Systems Biology (MSB), Ulm University, Ulm, Germany.

**Simon Laban** is a senior physician at the Department of Otorhinolaryngology, Head and Neck Surgery, Ulm University Medical Center, Germany.

**Axel Fürstberger** is a postdoctoral researcher at MSB and deputy of Core Unit Bioinformatics, Ulm University, Ulm, Germany.

**Silke D. Kühlwein** is a PhD student at MSB and the International Graduate School of Molecular Medicine, Ulm University, Germany.

**Nensi Ikonomi** is a PhD student at MSB and the International Graduate School of Molecular Medicine, Ulm University, Germany.

**Thomas K. Hoffmann** is Medical Director, Department of Otorhinolaryngology, Head and Neck Surgery, Ulm University Medical Center, Germany.

**Cornelia Brunner** is a research laboratory head at the Department of Otorhinolaryngology, Head and Neck Surgery, Ulm University Medical Center, Germany.

**Donna S. Neuberg** is a senior lecturer of Biostatistics at the Department of Biostatistics, Dana-Farber Cancer Institute, Boston, Massachusetts, USA.

**Verena Gaidzik** is a senior physician at the Department of Internal Medicine III, Ulm University Medical Center, Germany.

**Hartmut Döhner** is Medical Director, Department of Internal Medicine III, Ulm University Medical Center, Germany.

**Johann M. Kraus** is a postdoctoral researcher at MSB, Ulm University, Germany.

**Hans A. Kestler** is Director, Institute of Medical Systems Biology, and head of Core Unit Bioinformatics, Ulm University, Ulm, Germany, and an associated group leader at the Leibniz Institute on Aging–Fritz Lipmann Institute Jena, Jena, Germany.
