# Erratum to: Analysis, identification and visualization of subgroups in genomics

**DOI:** 10.1093/bib/bbab280

**Published:** 2021-07-06

**Authors:** Gunnar Völkel, Simon Laban, Axel Fürstberger, Silke D Kühlwein, Nensi Ikonomi, Thomas K Hoffmann, Cornelia Brunner, Donna S Neuberg, Verena Gaidzik, Hartmut Döhner, Johann M Kraus, Hans A Kestler

**Affiliations:** Institute of Medical Systems Biology (MSB), Ulm University, Ulm, Germany; Department of Otorhinolaryngology, Head and Neck Surgery, Ulm University Medical Center, Germany; MSB and deputy of Core Unit Bioinformatics, Ulm University, Ulm, Germany; International Graduate School of Molecular Medicine, Ulm University, Germany; International Graduate School of Molecular Medicine, Ulm University, Germany; Department of Otorhinolaryngology, Head and Neck Surgery, Ulm University Medical Center, Germany; Department of Otorhinolaryngology, Head and Neck Surgery, Ulm University Medical Center, Germany; Department of Biostatistics, Dana-Farber Cancer Institute, Boston, Massachusetts, USA; Department of Internal Medicine III, Ulm University Medical Center, Germany; Department of Internal Medicine III, Ulm University Medical Center, Germany; MSB, Ulm University, Germany; Institute of Medical Systems Biology, and head of Core Unit Bioinformatics, Ulm University, Ulm, Germany

In the originally published version of this manuscript, there was an error in co-author Thomas K. Hoffmann's name. The full name should read: “Thomas K. Hoffmann” instead of “Thomas K. Hoffman”. This error has now been corrected online.

