# Supplementary material for: Analysis, identification and visualization of subgroups in genomics
Source: Brief Bioinform. 2020 Sep 21;22(3):bbaa217. doi: 10.1093/bib/bbaa217 (PMC8138884; doi:10.1093/bib/bbaa217)
Supplement: Supplement_algorithm_bbaa217 [file supplement_algorithm_bbaa217.pdf]

Supplementary information

Multi-Objective Gene Selection

Problem Definition

The multi-objective gene selection problem consists of finding optimal subsets of genes from a given datasets with respect to multiple objectives, such as sample coverage, alteration overlap and subset size. Found subsets represent optimal trade-offs between the chosen objectives. The multi-objective gene selection problem is based on binary alteration data, such as mutation data, binarized expression data or binarized methylation data. For a given set of samples $\Theta = \{\theta_1, \dots \theta_m\}$ and a given set of genes $\mathcal{G} = \{g_1, \dots, g_n\}$, the set of samples with an alteration in $g \in \mathcal{G}$ is denoted as $\Gamma(g) \subseteq \Theta$. A sample $\theta \in \Theta$ is said to be covered by a gene $g \in \mathcal{G}$ if and only if the sample θ has an alteration in the gene g, written as $\theta \in \Gamma(g)$. A sample $\theta \in \Theta$ is covered by a set of genes $G \subseteq \mathcal{G}$ if and only if the sample is covered by one of the genes in G, i.e. $\theta \in \bigcup_{g \in G} \Gamma(g) = \Gamma(G)$. The coverage $\gamma(g)$ of a gene $g \in \mathcal{G}$ is the number of samples with an alteration in g, i.e. $\gamma(g) = |\Gamma(g)|$. The coverage $\gamma(G)$ of a set of genes $G \subseteq \mathcal{G}$ is the number of different samples that have alterations in at least one of the genes in G

$$\gamma(G) = \left|\Gamma(G)\right| = \left|\bigcup_{g \in G} \Gamma(g)\right| \quad (1)$$

and the overlap of G is defined as

$$\omega(G) = \left(\sum_{g \in G} \gamma(g)\right) - \gamma(G) \,. \quad (2)$$

This overlap definition accounts for the number of additional genes in G that cover the same sample in contrast to counting the number of samples covered by more than one gene from G. The gene selection task consists in finding a subset of genes $G \subseteq \mathcal{G}$ such that the number of covered samples $\gamma(G)$ is maximized and either the overlap $\omega(G)$ or the number of genes $|G|$ is minimized. There is no obvious choice for a weighting between the two optimization objectives. A setup using equal weights on unnormalized objectives has been used previously [1]. Hence, the described research questions are formulated as a multi-objective gene selection task that consists of finding a Pareto-optimal set $\mathcal{S}^* \subseteq \mathcal{S}$ of gene subsets within the set of all gene subsets $\mathcal{S} = \{G \subseteq \mathcal{G}\}$. The solutions, i.e. gene subsets, in the Pareto-optimal set $\mathcal{S}^*$ are the optimal trade-offs between the two optimization objectives [2]. Pareto-optimality is based on the dominance relation $\lhd$, i.e. a solution G dominates another solution G', denoted as $G \lhd G'$, if and only if G is at least as good as G' in all objectives and strictly better in at least one. Based on this, the Pareto-optimal set is defined as the set of solutions that are not dominated by any other solution:

$$\mathcal{S}^* = \left\{ G' \in \mathcal{S} \mid \nexists G \in \mathcal{S} : \, G \lhd G' \right\} . \quad (3)$$

Figure 1 shows a Pareto-optimal set for a multi-objective optimization task maximizing coverage and minimizing overlap. The solutions dominated by the Pareto-optimal solution G' are marked.

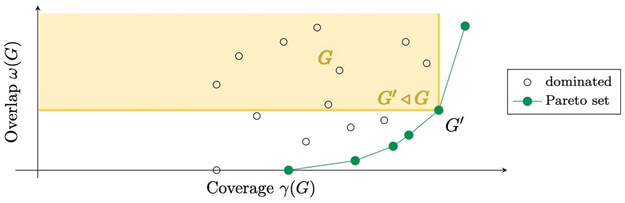

Fig. 1. Schematic Pareto set for a bi-objective optimization problem maximizing coverage and minimizing overlap. The solutions are shown as circles in a scatter plot of the two objectives coverage and overlap. The green-filled circles represent the Pareto-optimal solutions. The set of solutions (orange) dominated by the Pareto-optimal solution G' is shown.

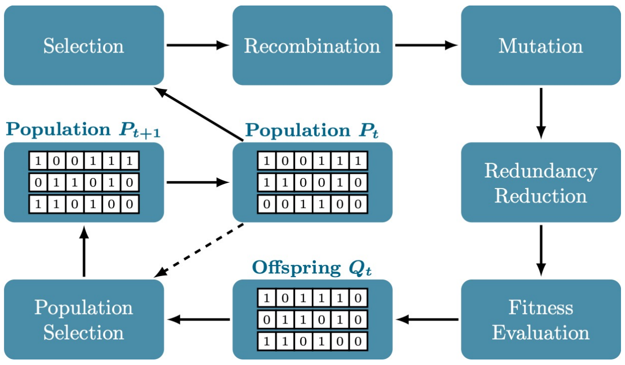

Fig. 2. Multi-objective evolutionary algorithm for the gene selection task. The steps of an iteration t during the algorithm are shown starting with the population P_t and resulting in a population P_{t+1} that will be processed in the next iteration $t+1$ provided that the specified computational budget is not exhausted. Solutions in the populations are represented as bit-strings.

Multi-Objective Evolutionary Algorithm

We developed an evolutionary algorithm for the multi-objective gene selection task based on the Non-dominated Sorting Genetic Algorithm II [2, NSGA-II] implemented by jMetal library v.5.3 [3]. This is a population-based metaheuristic which adapts concepts of the theory of evolution [4]. A set of solutions, called population, is evolved iteratively by applying recombination and mutation operators to the solutions (see Figure 2). The algorithm uses the following binary solution encoding. Genes are sorted by sample coverage in decreasing order. The i-th bit in a solution s encodes whether the i-th gene g is part of the gene set G encoded by this solution. Our NSGA-II for Multi-Objective Gene Selection is outlined in Algorithm 1. The algorithm starts with an initial population of $\mu \in \mathbb{N}$ randomly created solutions with a probability of $p_{\text{sel}} \in (0, 1)$ to set a bit to 1 (otherwise 0). Tournament selection with tournament size $\tau \in \mathbb{N}$ and the crowded-distance comparator are used to select solutions from the parent population for recombination and mutation. The crowded distance $d(s_i)$ of a solution s_i is calculated based on the permutations π_j resulting from sorting the solutions by the j-th objective f_j as follows:

$$d(s_i) = \sum_{j=1}^{k} \frac{f_j(s_{\pi_j(i+1)}) - f_j(s_{\pi_j(i-1)})}{f_j^{\max} - f_j^{\min}} \quad (4)$$

and $d(s_{\pi_j(1)}) = \infty$, $d(s_{\pi_j(\mu)}) = \infty$ for the extrema per objective f_j. With probability $p_{\text{cx}} \in [0, 1]$ a pair of selected solutions is modified by a single point crossover. The resulting solutions are

Algorithm 1: NSGA-II for Multi-Objective Gene Selection

$P_0 \leftarrow \{s_1, \ldots, s_\mu\}$ with random $s \in_{\mathcal{P}} \{0,1\}^n$ using probability $\mathcal{P}(X=1) = p_{\text{sel}}$
Evaluate population P_0
$t \leftarrow 0$
for $t \in \{1, \ldots, k\}$ **do**
  $P_t^{\text{mate}} \leftarrow$ `TournamentSelection`$(\tau,\, P_{t-1})$
  $Q_t \leftarrow$ `RedundancyReduction`(`Mutation`(p_{mut}, `Crossover`$(p_{\text{cx}},\, P_t^{\text{mate}})$))
  Evaluate offspring Q_t
  $P_t' \leftarrow$ `NonDominatedSortingSelection`(Q_t, P_{t-1})
return *non-dominated subset of* P_k

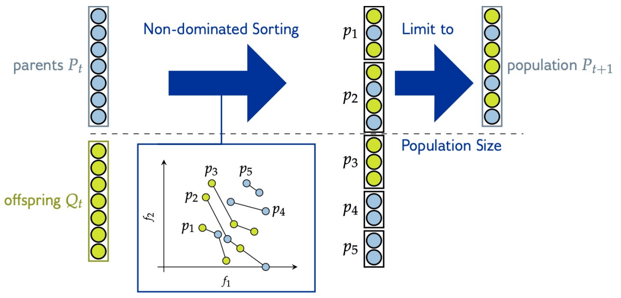

Fig. 3. Population selection based on non-dominated sorting and crowding distance. The parent and offspring solutions are sorted according to their occurrence in the non-dominanted sets of rank $1, 2, \ldots, r$. Complete non-dominated sets are selected for the next population of size μ with increasing rank. In case a non-dominated set cannot be selected completely, the solutions with the largest crowding distance are selected.

mutated using a bit-flip mutation with probability $p_{\text{mut}} \in (0,1)$. The objective values of the resulting offspring solutions are evaluated. The new population of μ solutions is determined by non-dominated sorting (see Figure 3) of the parent population and their offspring [2]. This is repeated for a specific number of iterations ($k \in \mathbb{N}$). The non-dominated solutions of the final population are the optimization results.

The evolutionary algorithm tends to have difficulties to eliminate genes quickly from the subset that have become redundant due to the addition of other genes. Hence, an additional redundancy reduction step is included after crossover and mutation to remove genes that only cover samples already covered by other genes. The redundancy of a gene is defined relative to a gene order. A gene g is considered redundant if the previous genes (G^{prev}) in the order already cover all the samples $\Gamma(g)$ that have an alteration in gene g, i.e.

$$\Gamma(g) \setminus \left(\bigcup_{g' \in G^{\text{prev}}} \Gamma(g') \right) = \emptyset\,. \quad (5)$$

Two gene orders are used to remove redundant genes from a solution: the order used in the bit-string encoding (decreasing sample coverage) and the corresponding reverse order (increasing sample coverages). Both orders are used to remove redundant genes from an offspring solution resulting in two solutions. From these two solutions the one with the better second objective, i.e. less redundancy, replaces the original offspring.

Objective Alteration Plots via Algorithmic Sorting

AVAtar facilitates the creation of objective and reproducible alteration plots by offering algorithmic sorting of genes and samples. The task of finding a gene order based on the additionally covered samples is formulated as a minimal Set Cover problem. A modified greedy algorithm [5] for Set Covering is applied (see Algorithm 2). Starting with an initial solution, the greedy algorithm incrementally adds the gene covering the most uncovered samples to its current partial solution, i.e. the gene g that maximizes the objective function $f(G,g) = \gamma(G \cup \{g\})$. For a gene order based on maximal coverage and maximal exclusivity (i.e. minimal overlap), this objective function is set to

$$f_\alpha^{\text{ex}}(G,g) = \alpha \cdot \gamma'(G \cup g) + (1-\alpha) \cdot (1 - \gamma'(G \cup g)) \rightarrow \max \quad (6)$$

including a weighting $\alpha \in [0,1]$ between coverage and exclusivity. To permit the weighting between both aims, the modified objective function uses relative coverage γ' and relative

Algorithm 2: Greedy Gene Sorting

$G_0 \leftarrow \emptyset$, $\mathcal{G}_1 \leftarrow \mathcal{G}$ and $t \leftarrow 1$
Empty sorted gene list π
while $\mathcal{G}_t \neq \emptyset$ **do**
  if $G_{t-1} = \emptyset$ **then** // *initial selection or restart after reaching maximal coverage?*
    $\gamma^{\max} \leftarrow \gamma(\mathcal{G}_t)$
    $(g_t, g_{t+1}) \leftarrow \arg\max \left\{ f_\alpha(\{g\}, g') \mid (g,g') \in \mathcal{G}_t^2,\, \gamma(g) > \gamma(g') \right\}$
    $\pi_t \leftarrow g_t$ and $\pi_{t+1} \leftarrow g_{t+1}$
    $G_t \leftarrow G_{t-1} \cup \{g_t\}$ and $G_{t+1} \leftarrow G_t \cup \{g_{t+1}\}$
    $\mathcal{G}_{t+1} \leftarrow \mathcal{G}_t \setminus \{g_t\}$ and $\mathcal{G}_{t+2} \leftarrow \mathcal{G}_{t+1} \setminus \{g_{t+1}\}$
    $t \leftarrow t+2$
  else
    $g_t \leftarrow \arg\max \left\{ f_\alpha(G_{t-1}, g) \mid g \in \mathcal{G}_t \right\}$
    $\pi_t \leftarrow g_t$
    $\mathcal{G}_{t+1} \leftarrow \mathcal{G}_t \setminus \{g_t\}$
    if $\gamma(G_t) < \gamma^{max}$ **then**
      $G_t \leftarrow G_{t-1} \cup \{g_t\}$
    else
      $G_t \leftarrow \emptyset$ // *maximal coverage reached* $\rightarrow$ *restart*
    $t \leftarrow t+1$
return π

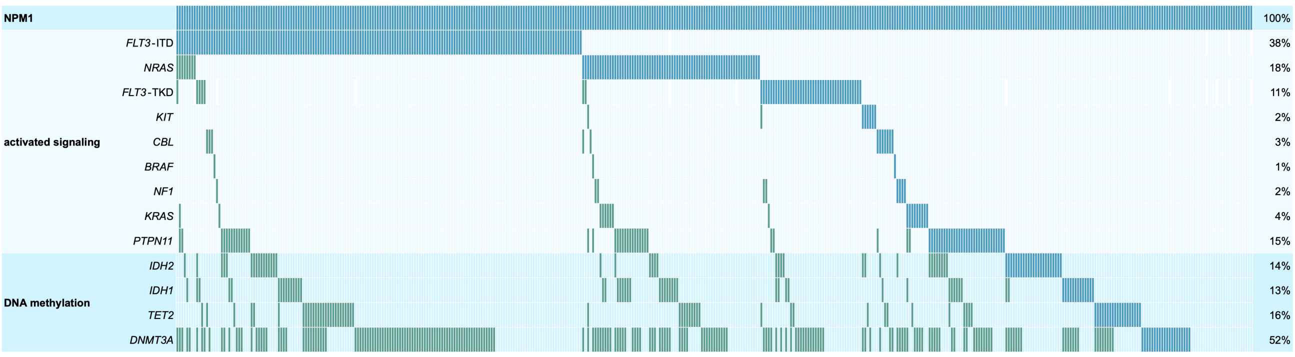

Fig. 4. Visualization for genomic data of a subset of $1,540$ patients from our AML dataset [6]. Genes are grouped into predefined functional categories. In this subgroup of NPM1 mutated patients, most co-mutations occur in genes associated to the functional groups *activated signaling* and *DNA methylation*. NPM1 is fixed to the first position and the remaining genes are sorted according to a trade-off between mutually exclusivity and coverage using $\alpha = 0.4$. The sorted alteration plot highlights the molecular heterogeneity across patients. This visualization with AVAtar shows the molecular heterogeneity within these patients clearly.

overlap ω' of the gene set $G \subseteq \mathcal{G}$ compared to the set of all genes $\mathcal{G}$ defined as

$$\gamma'(G) = \frac{\gamma(G)}{\gamma(\mathcal{G})} \quad \text{and} \quad \omega'(G) = \frac{\omega(G)}{\omega(\mathcal{G})} \,. \tag{7}$$

Similarly, sorting genes according to co-occurring alterations and coverage can be achieved with the following objective function:

$$f_\alpha^{\text{co}}(G, g) = \alpha \cdot \gamma'(G \cup g) + (1 - \alpha) \cdot \gamma'(G \cup g) \to \max \,. \tag{8}$$

Finally, samples are sorted lexicographically according to the gene order. Figure 4 shows a visualization for genomic data of a subset of $1,540$ patients from an AML dataset [6] using AVAtar’s ability to additionally group genes on predefined functional categories. In this subgroup of NPM1 mutated patients, most co-mutations occur in genes associated to the functional groups *activated signaling* and *DNA methylation*. NPM1 is fixed to the first position and the remaining genes are sorted according to a trade-off between mutually exclusivity and coverage using $\alpha = 0.4$. The sorted alteration plot highlights the molecular heterogeneity across patients.

References

[1] Vandin, F., Upfal, E. and Raphael, B. J. De novo discovery of mutated driver pathways in cancer. *Genome Res*, 2012; **22**:375–385.

[2] Deb, K. Multi-Objective Optimization Using Evolutionary Algorithms. NY, USA: John Wiley & Sons, Inc., 2001.

[3] Nebro, A. J., Durillo, J. J. and Vergne, M. Redesigning the jMetal Multi-Objective Optimization Framework. In: Proceedings of the Companion Publication of the 2015 Annual Conference on Genetic and Evolutionary Computation, GECCO Companion ’15. NY, USA: ACM, 1093–1100.

[4] Reeves, C. R. Handbook of Metaheuristics, chapter Genetic Algorithms. Springer US, 2010; 109–139.

[5] Cormen, T. H., Leiserson, C. E., Rivest, R. L. *et al.* Introduction to Algorithms. The MIT Press, 3rd edition, 2001.

[6] Papaemmanuil, E., Gerstung, M., Bullinger, L. *et al.* Genomic classification and prognosis in acute myeloid leukemia. *N Engl J Med*, 2016; **374**:2209–2221.
